# Immunodeficiency in a Child with Rapadilino Syndrome: A Case Report and Review of the Literature

**DOI:** 10.1155/2015/137368

**Published:** 2015-05-06

**Authors:** M. M. G. Vollebregt, A. Malfroot, M. De Raedemaecker, M. van der Burg, J. E. van der Werff ten Bosch

**Affiliations:** ^1^Department of Pediatrics, University Hospital Brussels, 1090 Brussels, Belgium; ^2^Department of Genetics, University Hospital Brussels, 1090 Brussels, Belgium; ^3^Department of Immunology, Erasmus MC, 3015 CN Rotterdam, Netherlands; ^4^Department of Pediatric Hematology, Oncology and Immunology, University Hospital Brussels, 1090 Brussels, Belgium

## Abstract

Rapadilino syndrome is a genetic disease characterized by a characteristic clinical tableau. It is caused by mutations in RECQL4 gene. Immunodeficiency is not described as a classical feature of the disease. We present a 2-year-old girl with Rapadilino syndrome with important lymphadenopathies and pneumonia due to disseminated *Mycobacterium lentiflavum* infection. An immunological work-up showed several unexpected abnormalities. Repeated blood samples showed severe lymphopenia. Immunophenotyping showed low T, B, and NK cells. No Treg cells were seen. T cell responses to stimulations were insufficient. The IL12/IL23 interferon gamma pathway was normal. Gamma globulin levels and vaccination responses were low. With this report, we aim to stress the importance of screening immunodeficiency in patients with RECQL4 mutations for immunodeficiency and the need to further research into its physiopathology.

## 1. Introduction

Rapadilino syndrome (RS) is a genetic disease with a characteristic clinical tableau. The name is an acronym standing for radial (hypo)aplasia, patellae (hypo)aplasia and cleft or highly arched palate, diarrhoea and dislocated joints, little size and limb malformation, and nose slender and normal intelligence [1]. Like Rothmund-Thomson syndrome (RTS) and Baller-Gerold syndrome (BGS), the syndrome is caused by mutations in* RECQL4* gene. This gene encodes a protein that plays a role in the initiation of DNA replication as well as in DNA repair. Immunodeficiency has not been described as a prominent clinical feature in any of the 3 syndromes. RTS is a rare autosomal recessively inherited genodermatosis with a heterogeneous clinical presentation. It is characterized by a characteristic facial rash appearing in infancy (poikiloderma), short stature, radial ray defects, variable degree of osteopenia, sparse scalp hair, eyelashes, and eyebrows, dental abnormalities, and cataract. Moreover, RTS patients are at increased risk of cancer, especially osteosarcoma and nonmelanoma skin cancer, but also leukemia and a range of others tumors [2]. RTS is a very rare disease and reliable data on its prevalence are not available. To date, approximately 300 patients have been recorded in the medical literature [3]. BGS is characterized by a combination of coronal craniosynostosis, manifesting as abnormal shape of the skull (brachycephaly) with ocular proptosis and bulging forehead, and radial ray defect, manifesting as oligodactyly (reduction in number of digits), aplasia or hypoplasia of the thumb, and/or aplasia or hypoplasia of the radius. The prevalence of BGS is unknown; it is probably less than 1 : 1.000.000 [4].

We present a now 4-year-old girl diagnosed with RS presenting with significant lymphadenopathies and pneumonia due to disseminated* Mycobacterium lentiflavum* infection. An immunological work-up showed several unexpected abnormalities. The child was treated and the clinical condition gradually improved. We suggest screening children with RECQL4 mutations for immunodeficiency and stress the need for further research into its physiopathology.

## 2. Case Report

**Figure 1:**
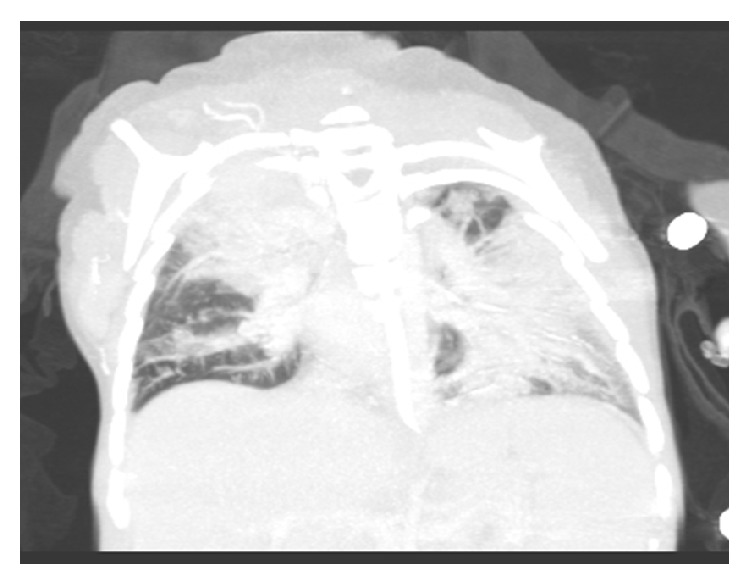
CT scan showing mediastinal enlargement and bilateral infiltrates.

A 2-year-old girl was admitted because of severe lymphadenopathies. She had been diagnosed with RS at birth. No important infections occurred in the first years of life until these unexplained lymphadenopathies. Because an increased risk of lymphoma at a young age has been documented in patients with RS [6], a biopsy was taken, excluding a malignancy. For the 8 months that followed, the girl was lost to follow-up in our center, but she represented later that year with cough, fever, and dyspnea requiring oxygen. Lymphadenopathies persisted in all regions. There were no signs of hepatosplenomegaly. Chest X-ray showed mediastinal enlargement and bilateral infiltrates (Figure 1).

**Figure 2:**
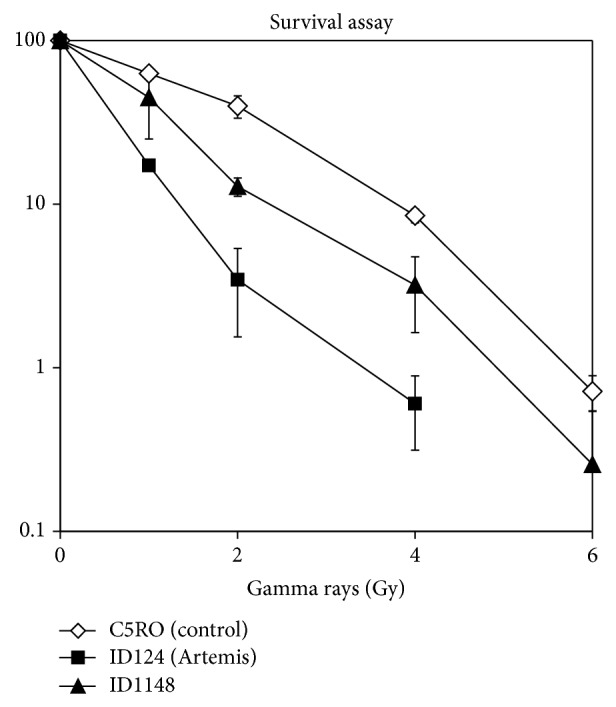
Radiosensitivity assay. Clonogenic survival assays with primary skin were performed as described in Noordzij et al., Blood 2003 [5]. In short, primary skin fibroblasts in exponential growth were trypsinized, and 1000–2,000 cells (10,000–20,000 cells for the highest doses) were seeded into 10 cm plastic dishes (2 dishes per dose) and irradiated at room temperature with 0, 1,2,4 or 6 Gy. After 12–14 days, the cells were rinsed with 0.9% NaCl and stained with 0.25% methylene blue for survival assessment. Two independent survival experiments were performed.

Cultures from bronchoalveolar lavage remained negative for bacteria, including mycobacteria. PCR for viruses (CMV, EBV) and mycoplasma were negative. Because a slight lymphopenia was observed in the routine blood sample, an immunological work-up was performed (Table 1). Hypogammaglobulinemia was observed. Antibodies against the received childhood vaccinations (pneumococcus, tetanus, rubella, polio, and hepatitis B) were all negative. Revaccination with Pneumo 23 and tetanus did not lead to an increase in the antibody titers. T cell numbers were low, with a slightly diminished function. The number of CD4+CD25+FoxP3+ regulatory T cells was remarkably low (Table 1). Switched memory B cells were slightly low according to the Euroclass criteria [7]. The number of double negative T cells, vitamin B12, and Fas mediated apoptosis were normal. The interferon gamma/interleukin 23 pathway was intact. Expression of IL-12 receptor beta 1 and IFN-gamma receptor expression were analysed by flow cytometry. The production of IFN-gamma was measured after stimulation of white blood cells with phytohaemagglutinin and staphylococcal enterotoxin B. HIV screening was negative. Radiosensitivity was mildly increased (Figure 2).

A lymph node biopsy showed signs of follicular and interfollicular hyperplasia as well as granulomas. Ziehl-Nielssen staining and IGRA (Interferon Gamma Release Assay) test were negative. PCR for CMV and EBV were negative as were cultures for bacteria. Finally, a culture from the bone marrow became positive for* Mycobacterium lentiflavum*. The child was treated accordingly and gradually improved, although the lymphadenopathies persisted.

Because of the poor responses to vaccination, the child is receiving intravenous immunoglobulin substitution therapy. Until now, the patient is still dependent on immunoglobulin substitution therapy. Moreover, she now receives* Pneumocystis jiroveci* prophylaxis. Ionizing radiation is used as little as possible. Under these circumstances, the child is doing well. No more invasive infections were observed so far.

## 3. Discussion

RS, RTS, and BGS are caused by mutations in* RECQL4* gene. The RecQ family of helicases is a group of proteins that play a role in genomic stability. The family contains 5 members, 3 of which are involved in disorders characterized by genomic instability. RecQL4 seems to be involved in more than one cellular pathway involved in DNA repair, but the exact function is not well understood [8]. There is evidence that RECQL4 plays multiple key roles in DNA metabolism, as it is involved in single-stranded DNA annealing activity, DNA replication, double strand break repair, and repair of UV or ionizing radiation induced DNA damage [2]. How defects in these proteins can lead to such a broad spectrum of clinical manifestations needs further to be investigated. How defects in this protein lead to immunodeficiency is even less clear [9].

Immunodeficiency is a well described feature of other chromosomal breakage syndromes such as ataxia telangiectasia (AT) and Bloom syndrome. In Bloom syndrome, the clinical phenotype is variable, with prolonged panhypogammaglobulinaemia, severe respiratory infection causing chronic lung disease, and sinopulmonary infection being the most common manifestations. In AT, the immunodeficiency is characterized by both cellular and humoral impairment, but clinical manifestations are extremely variable, ranging from normal to profoundly reduced responses to bacterial antigens. Recurrent sinopulmonary infection is common and is associated with hypogammaglobulinaemia due to B cell maturation defects. Cellular immunodeficiency is characterized by defective thymic development, with macroscopic absence of the thymus at postmortem examination. Another hypothesis can be postulated in a defective class switch mechanism. Class switch recombination is known to be defective in other DNA repair syndromes and could be deficient in lymphocytes from patients with RS [10]. The fact that the patient had low switched memory B cells could be compatible with this hypothesis but further research will have to show whether or not this hypothesis is correct.

There are only few data on the quality of the immune system in children with RECQL4 mutations. There is an increasing number of reports on increased susceptibility to infections and immunodeficiency in RTS [10–14]. Our patient presented with an intriguing immunological phenotype, which was very similar to the phenotype described by de Somer et al. [2]. Our patient had low T and B as well as a low percentage of switched memory B cells and NK cells. The patient with RTS described by de Somer et al. [2] also revealed by immunophenotyping low T and NK cells and a low number of class switched B cells and diminished specific antibody response. The low T cells might be partially due to diminished thymic output. RECQL4 is highly expressed in the thymus and KO mice have smaller thymi, suggesting a role for RECQL4 in T cell development [15]. The finding that regulatory T cells were absent in our patient is intriguing, although it is unclear if this phenomenon is typical for patients with Recq4 mutations or a result of the severe infection. The finding that in Mycobacterium tuberculosis infection regulatory T cells are high instead of low makes the second hypothesis less probable [16]. Further research would help to clarify this issue.

The diminished thymic output cannot explain the defects in the other lymphocytes nor the low percentage of switched memory B cells. This finding is observed in AT patients as well. Specific antibodies against poliovirus, measles, and hepatitis B virus were lacking in our patient, as in the patient described by de Somer et al. [2], despite adequate immunization. Anti-pneumococcal antibody response to vaccination with the polysaccharide pneumococcal vaccine was also low.

Granulomas in a variety of organs are not uncommon in patients with a wide range of immunodeficiencies of different origins, [17, 18] as well as in some infections, such as mycobacteria. In the group of patients with DNA repair problems, this phenomenon has been described as well [2, 19]. This finding could thus be interpreted as a sign of the underlying infection and further support the diagnosis of an immunodeficient state.

Although our patient had no antibodies against the pneumococcal vaccinations she had received, she did not suffer from recurrent ear infections or other pneumococcal infections. In contrast, her presenting infection, disseminated* Mycobacterium lentiflavum*, has been the only important infection in this patient. Disseminated mycobacterial infections usually point to a defect in the Interferon gamma/IL12/23 pathway, which was normal in our patient. This further stresses the need for further investigation in these patients to try to determine the exact mechanism leading to this immunodeficiency.

This case report suggests that immunodeficiency can occur in children with REQL4 mutations and that immunological screening should be performed as a standard of care. RTS, RS, and BGS are genetically related disorders with mutations in the same gene but each a partly overlapping but distinct clinical phenotype. In literature, a number of reports on increased susceptibility to infections or abnormalities in the immune system of patients with RTS have appeared recently [10–14]; also one case of a patient with BGS is reported [20]. But so far, this has not been reported in patients with RS. Larger studies will be necessary to conclude if the immunological abnormalities found in this patient are indeed common in children with RS as well.

## Figures and Tables

**Table 1:** Immunological work-up.

			Normal values
White blood cells	4,7 × 10^3^/mm^3^		4,0–10,0 × 10^3^/mm^3^

Neutrophils	2,8 × 10^3^/mm^3^		1,5–8,5 × 10^3^/mm^3^

Lymphocytes	1,0 × 10^3^/mm^3^		2,3–5,6 × 10^3^/mm^3^

Immunoglobulin G	2,5 g/L		4,0–11,0 g/L

Immunoglobulin A	1,01 g/L		0,1–1,6 g/L

Immunoglobulin M	0,75 g/L		0,5–1,8 g/L

CD3+	411/mm^3^		900–4500/mm^3^

CD4+	279/mm^3^		500–2400/mm^3^

CD4RA	140/mm^3^		

CD4RO	139/mm^3^		

CD8	124/mm^3^		300–1600/mm^3^

CD8RA	52/mm^3^		

CD8RO	72/mm^3^		

CD25+CD127−FoxP3+	0		

CD19	84/mm^3^		200–1300/mm^3^

CD3−CD16+CD56+	5/mm^3^		100–1000/mm^3^

Response of T cells to PHA	low		

	Before vaccination	After vaccination	Normal values

Anti-pneumococcal AB	<3 IE/mL	<3 IE/mL	>19/mL

Anti-tetanos AB	<0.01 IE/mL	0.1 IE/mL	>0.1 IE/mL

Anti-poliovirus AB	Absent	Present	

Anti-rubella AB	300 IE	—	Positive

Anti-mumps AB	Negative	—	Positive
